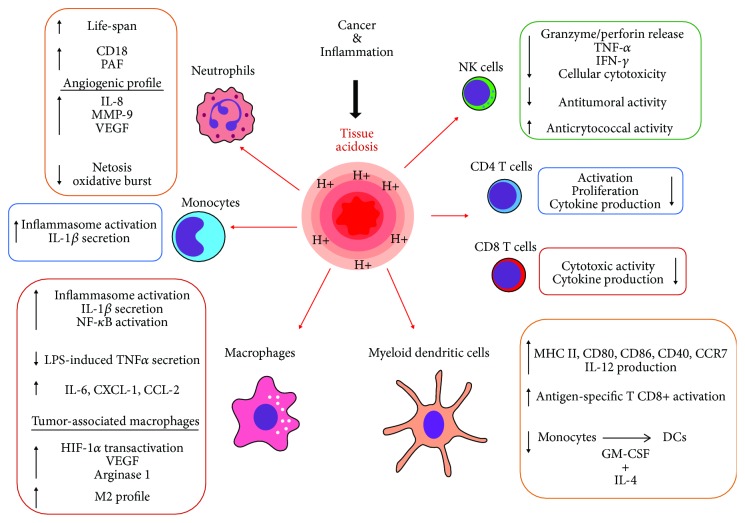# Unravelling the Interplay between Extracellular Acidosis and Immune Cells

**DOI:** 10.1155/2018/1218297

**Published:** 2018-12-30

**Authors:** Fernando Erra Díaz, Ezequiel Dantas, Jorge Geffner

**Affiliations:** Instituto de Investigaciones Biomédicas en Retrovirus y SIDA (INBIRS), Universidad de Buenos Aires, CONICET, Ciudad de Buenos Aires, Argentina

## Abstract

The development of an acidic tissue environment is a hallmark of a variety of inflammatory processes and solid tumors. However, little attention has been paid so far to analyze the influence exerted by extracellular pH on the immune response. Tissue acidosis (pH 6.0 to 7.0) is usually associated with the course of infectious processes in peripheral tissues. Moreover, it represents a prominent feature of solid tumors. In fact, values of pH ranging from 5.7 to 7.0 are usually found in a number of solid tumors such as breast cancer, brain tumors, sarcomas, malignant melanoma, squamous cell carcinomas, and adenocarcinomas. Both the innate and adaptive arms of the immune response appear to be finely regulated by extracellular acidosis in the range of pH values found at inflammatory sites and tumors. Low pH has been shown to delay neutrophil apoptosis, promoting their differentiation into a proangiogenic profile. Acting on monocytes and macrophages, it induces the activation of the inflammasome and the production of IL-1*β*, while the exposure of conventional dendritic cells to low pH promotes the acquisition of a mature phenotype. Overall, these observations suggest that high concentrations of protons could be recognized by innate immune cells as a danger-associated molecular pattern (DAMP). On the other hand, by acting on T lymphocytes, low pH has been shown to suppress the cytotoxic response mediated by CD8+ T cells as well as the production of IFN-*γ* by TH1 cells. Interestingly, modulation of tumor microenvironment acidity has been shown to be able not only to reverse anergy in human and mouse tumor-infiltrating T lymphocytes but also to improve the antitumor immune response induced by checkpoint inhibitors. Here, we provide an integrated view of the influence exerted by low pH on immune cells and discuss its implications in the immune response against infectious agents and tumor cells.

## 1. Introduction

Extracellular acidosis is a hallmark of inflammatory processes. Accumulation of protons in the extracellular space is frequently associated with the course of inflammatory responses against bacteria in peripheral tissues, where pH values as low as 5.5 have been described [1–6]. In this scenario, local acidosis appears to be induced by three major factors: (1) tissue hypoxia caused by the damage of small blood vessels and the metabolic activity of infiltrating leukocytes, resulting in a switch towards glycolytic metabolism and the subsequent accumulation of lactic acid [7–12]; (2) the massive production of protons by neutrophils during the activation of the respiratory burst [13–15]; and (3) the accumulation of short-chain fatty acids produced by bacteria [16–18]. Autoimmune and allergic diseases are also associated to the accumulation of protons in the extracellular space at the sites of tissue injury. Analysis of compromised joints in patients with rheumatoid arthritis revealed low pH values in the synovial fluid being acidosis associated to both synovial fluid leukocytosis and radiological joint destruction [19–21]. On the other hand, observations made in samples of exhaled breath condensate from asthmatic patients revealed that acute asthma exacerbations are associated to the acidification of the airway [22–24]. Similar findings were made in patients with acute lung injury [25].

A large body of evidence indicates that acidosis and hypoxia are hallmarks of tumors as well as crucial determinants of tumor progression. Observations made in solid tumors such as malignant melanomas, brain tumors, sarcomas, breast cancer, squamous cell carcinomas, and adenocarcinomas showed that tumor microenvironments reach pH values ranging from 5.8 to 7.4 [26–34]. Tumor cells usually show a highly glycolytic metabolism leading to the decrease in the pH of the tumor microenvironment due to lactic acid production. The Warburg effect, identified by Otto Warburg and colleagues in the 1920s, describes the elevated rate of glucose uptake and preferential production of lactate by tumor cells, even in the presence of oxygen. More recent studies, however, suggest that lactate production by tumor cells also involves the metabolism of nonglucose substrates [35–37]. DeBerardinis et al. demonstrated that glutamine can be metabolized in cancer cells through the citric acid cycle and that this pathway represents a major source of lactate [35]. It is not only cancer cell metabolism that contributes to the acidification of the tumor microenvironment. Additionally, the poor and disorganized tumor vasculature prevents an efficient washout of protons from the extracellular space leading to the acidification of the extracellular space [33, 34, 38–40]. Interestingly, extracellular acidosis has been shown to favor cancer progression by promoting local tumor invasion and also distant metastatic spread [27]. Moreover, different strategies have been explored to exploit the relative acidity of tumor versus normal tissue in order to improve the efficacy of antitumor chemotherapy [27, 39–42].

In spite that low pH is a common feature in inflammatory environments and tumors, little attention has been paid to determine how the function of immune cells is modulated by changes in the extracellular pH. This review summarizes our current understanding of the immunomodulatory actions induced by low pH on the course of the innate and adaptive immune response and discusses the mechanisms involved, as well as the feasibility of using therapeutic strategies targeting extracellular acidosis.

## 2. Sensing of Protons by Immune Cells

Mammalian cells are able to sense changes in the pH of the extracellular medium, particularly, the accumulation of protons in the extracellular space. However, the mechanism underlying the sensing of protons and the signaling pathways activated by them are poorly characterized. The emerging field of pH sensors expressed on the cell surface has shed some light about how cells recognize and respond to protons [43, 44]. G protein-coupled receptors (GPCRs), including GPR68 (OGR1), GPR65 (TDAG8), GPR4, and GPR132 (G2A) are widely expressed by immune cells. They act as receptors for lysolipids such as sphingosylphosphorylcholine, lysophosphatidylcholine, and psychosine, but they are also activated by low pH (range 6.4 to 6.8) through the protonation of the imidazole side chain of histidine residues (pI < 6.5) located in the extracellular domain of these receptors. This results in different signaling mechanisms mediated by the activation of phospholipase C (PLC), the induction of Ca^2+^ transients, the stimulation of adenylyl cyclase which leads to the synthesis and accumulation of cAMP, and the activation of the protein kinase A/ERK-signaling pathway [45–48]. Acid-sensing ion channels (ASICs) represent a second group of receptors activated by protons (half-maximal activation at pH < 6.7) [49–52]. They are cation-selective ion channels involved in pain perception, ischemic stroke, mechanosensation, learning, and memory [53]. ASICs comprise six isoforms (ASIC1a, ASIC1b, ASIC2a, ASIC2b, ASIC3, and ASIC4) mainly expressed in peripheral sensory and central nervous system neurons [53]. The pattern of ASIC expression in immune cells has not been clearly defined; however, these receptors have been shown to be expressed in monocytes, macrophages, and dendritic cells [54–56]. A third family of proton receptors includes the transient receptor potential channel vanilloid subfamily 1 (TRPV1). They are cation channels activated by low pH (~6.0), resulting in Ca^2+^ influx [57]. Originally associated with sensory neurons, TRPV1 has been shown to be highly expressed in immune cells [58–60]. Activation of TRPV1 induces the influx of Ca^2+^ and the subsequent activation of a number of transcription factors, such as NF-*κ*B and NFAT [61, 62].

There are also mechanisms through which protons might modulate cellular responses without interacting with cell surface receptors. Extracellular acidosis has been shown to be capable of lowering cytosolic pH in immune cells through the rapid diffusion into the cell of CO_2_ originating from the reaction of protons with extracellular bicarbonate, the subsequent hydration of CO_2_, and the overproduction of intracellular protons [63, 64]. In fact, intracellular acidification has been shown to promote different responses such as neutrophil shape change and chemotaxis, as well as the production of IL-1 by mononuclear phagocytes [65–68]. It should be noted, however, that the relative contribution of receptor-dependent and independent mechanisms in the immunomodulatory effects induced by low pH remains to be further clarified.

## 3. Innate Immune Cells and Low pH

There are a number of studies, mostly performed in vitro, analyzing the influence exerted by low pH on the function of innate immune cells. Some contradictory results have been reported perhaps reflecting distinct approaches used to lower the pH of the culture medium. While some studies have used isotonic solutions of HCl, others have employed lactic acid, the final product of anaerobic glycolysis. Lactate, which has long been merely considered as a bystander product of cell metabolism, is able to exert by itself important immunomodulatory effects [69]. Thus, it was not unexpected to find different observations by using HCl or lactic acid to change the pH of the culture medium. Those studies that have investigated both compounds in concert support the notion that low pH itself exerts major immunomodulatory effects and also exacerbates lactate-mediated effects [70].

### 3.1. Neutrophils

Neutrophils play a critical role in host defense against bacterial and fungal infections and are also involved in the pathogenesis of a number of inflammatory conditions [71]. Extracellular acidosis has been shown to induce either stimulatory or inhibitory effects on neutrophil responses, depending on the function analyzed. Most of the published studies were performed using human neutrophils. Early reports showed that both oxygen consumption and O_2_^−^ production induced by different stimuli such as the chemotactic peptide fMLP, opsonized zymosan, and PMA are markedly inhibited when assessed in citrate-phosphate buffer adjusted to acidic values of pH (range 6.0 to 7.0) [72]. Consistent with these observations, more recent studies have shown that neutrophils suspended in CO_2_-bicarbonate-buffered medium adjusted to low pH values show a reduced ability to produce extracellular traps (NETs) but a higher ability to kill phagocytized bacteria [73]. On the other hand, studies performed by us and other groups have shown that low pH induces neutrophil shape change, a transient increase in cytosolic Ca^2+^ levels, the upregulation of cell surface expression of CD18, and the production of platelet-activating factor (PAF) via activation of phospholipase A2 [67].

We have previously reported that extracellular acidosis induces both a fall in intracellular pH (pHi) and a delay of neutrophil apoptosis, suggesting that extracellular acidosis might delay apoptosis by reducing pHi [67]. Interestingly, we also observed that fever-range hyperthermia accelerates the rate of neutrophil apoptosis at neutral pH but markedly increases neutrophil survival induced by low pH [74]. Because previous reports have shown that hyperthermia promotes intracellular acidification in tumor cells by inhibiting the Na^+^/H^+^ antiporter, we hypothesized that the prosurvival effect induced by hyperthermia at low pH values could be related to its ability to decrease pHi. We found that hyperthermia did not decrease the pHi of neutrophils cultured at neutral pH values, but it significantly decreased the pHi of neutrophils cultured at acidic pH values. Moreover, we found that two Na^+^/H^+^ exchanger inhibitors reproduced the antiapoptotic effect induced by hyperthermia, suggesting that it delays neutrophil apoptosis by inhibiting the Na^+^/H^+^ antiporter further decreasing pHi. Of note, while the prolongation of neutrophil survival induced by pathogen-associated molecular patterns (PAMPs), danger-associated molecular patterns (DAMPs), and inflammatory cytokines is usually associated to the preservation of classical neutrophil effector functions such as phagocytosis and reactive oxygen species (ROS), the antiapoptotic effect induced by low pH and hyperthermia promoted an alternative functional profile characterized by a poor phagocytic ability, a very low production of ROS, a very high expression of the *β*2 integrin CD11b/CD18, and a high ability to suppress T cell responses and to produce the angiogenic factors IL-8, VEGF, and the matrix metallopeptidase 9 (MMP-9) [74]. These observations suggest that acting together, local acidosis and fever might promote neutrophil differentiation into a profile similar to that described for tumor-associated neutrophils (TANs). Neutrophils make up a significant fraction of the inflammatory cell infiltrate found in a large variety of tumors [75], and clinical studies involving different tumors have shown that TANs confer a poor prognosis in cancer patients [76–78]. Tumor-associated neutrophils seem to contribute to cancer growth and metastasis through different mechanisms, such as suppression of T cell function, production of angiogenic factors, and secretion of proteases, among them elastase and MMP-9 [75]. This functional profile closely resembled the profile observed for neutrophils cultured at low pH values and febrile-range temperatures [74].

### 3.2. Monocytes and Macrophages

The function of monocytes and macrophages is also regulated by changes in the pH of the extracellular medium. Most of the studies performed in this field have analyzed the influence exerted *in vitro* by low pH on the production of cytokines by human, murine, rat, and rabbit macrophages [68, 79–83]. Low pH (6.0 to 6.5) has been shown to induce the activation of the inflammasome and the production of IL-1*β* by human monocytes and macrophages [68, 83]. Additional experiments suggest that the stimulation of IL-1*β* production is promoted by a drop in pHi, and not through the interaction of extracellular protons with cell surface receptors. In fact, the inhibition of the major regulators of intracellular pH such as the plasmalemmal V-type H+ ATPases and the Na^+^/H^+^ exchangers, which extrude intracellular protons, resulted not only in a drop of pHi but also in the promotion of IL-1*β* secretion [68, 83].

It was also reported that lactic acidosis, but not low pH, stimulates the production of IL-23 by mononuclear phagocytes, promoting the development of a TH17 profile [84]. On the other hand, studies performed with rat peritoneal macrophages showed that mildly acidic conditions (pH 6.8-7.0) induce NF-*κ*B activation, the production of TNF-*α*, and the expression of the inducible form of nitric oxide synthase (iNOS) [79]. Contrasting with this observation, it has been reported that low extracellular pH (range 6.5-7.0) inhibits the production of TNF-*α* by rabbit alveolar macrophages [80], and consistent with this finding it has also been shown that either low pH or lactic acid markedly inhibits TNF-*α* production by human monocytes [85]. Interestingly, *in vivo* experiments made in mouse models of acute pneumonia and peritonitis induced by *Pseudomona aeruginosa* infection showed that acidosis stimulates the production of inflammatory cytokines, including IL-1*β*, IL-6, CXCL1, and CCL2, as well as the recruitment of neutrophils in the injured tissue [86, 87]. On the other hand, studies focused on tumor-associated macrophages (TAMs) have shown that lactic acidosis induces not only the expression of vascular endothelial growth factor (VEGF) but also the acquisition of a M2-like phenotype by TAMs [88]. These effects are mediated through the stimulation of the transcription factor hypoxia-inducible factor 1*α* (HIF-1*α*). Consistent with these observations, it was reported that low pH decreases gene expression of proinflammatory M1 markers such as iNOS, monocyte chemoattractant protein-1 (MCP1), and IL-6 in M1 macrophages, while it increased gene expression of M2 markers such as mannose receptor C-type 1 (MRC1), arginase 1 (ARG1), and chitinase-3-like protein in M2 macrophages [89]. These observations suggest that a macrophage growing at low pH undergoes an M1 to M2 phenotypic switch which might contribute to tumor growth.

### 3.3. Natural Killer Cells

Natural killer (NK) cells play an important role in the innate host defense against viruses and other intracellular pathogens as well as in antitumor immunity. There is a general agreement that extracellular acidosis inhibits the antitumoral activity of NK cells. Using unstimulated peripheral blood mononuclear cells (PBMCs), isolated NK cells, NK cell lines, and lymphokine-activated killer cells (LAK cells), it was shown that the release of perforin/granzyme-containing granules, the secretion of IFN-*γ* and TNF-*α*, and the cytotoxic response against tumor cells assessed in vitro were markedly inhibited at low pH values, in the range of 5.8 to 7.0 [90–92]. A similar inhibition of NK cell function was shown to be induced by lactic acid. Lactate dehydrogenase-A (LDH-A), the enzyme responsible for conversion of pyruvate to lactate, is highly expressed in tumor cells. Using a pancreatic cancer model, Husain et al. [93] reported that lactate from cancer cells inhibits NK cell activity in vivo and increases tumor size. Moreover, experiments performed in vitro showed that lactate inhibits NK cell cytotoxicity and that this suppressive effect was further amplified when assays were performed at low pH values. Consistent with these observations, it was also reported that lactic acid accumulation in melanomas inhibits tumor surveillance by NK cells. In fact, observations made in C57BL/6 mice showed that tumors with reduced lactic acid production developed slower than control tumors and showed an increased infiltration with IFN-*γ* producing T and NK cells [94].

Moreover, extracellular acidosis has been shown to prevent the generation of LAK cells induced by IL-2 [95]. Interestingly, experiments performed in vivo in a lymphoma mouse model demonstrated not only that tissue acidosis compromises the production of IFN-*γ* by NK cells but also that systemic alkalinization by oral delivery of bicarbonate increases the production of IFN-*γ* as well as the infiltration of tumor tissue by NK cells, delaying tumor growth [96]. Surprisingly, it has been reported that an acidic microenvironment does not suppress, but rather increases the killing of different species and strains of *Cryptococcus* mediated by NK cells [97]. This enhancing effect was shown to be associated to the stimulation of ERK1/ERK2 phosphorylation and the enhancement of perforin release. The reasons underlying the differences between the effects induced by low pH on the antitumoral and anticryptococcal activity of NK cells remain to be clarified. In this regard, it should be considered that low pH enhances *Cryptococcus* replication, and NK cells have been shown to kill the faster replicating *Cryptococcus* more efficiently than the slower replicating organisms. On the other hand, differences in the mechanisms responsible for the recognition of tumor cells and fungi should also be considered. In fact, while NK cells require the participation of LFA-1 for binding and killing of tumor cells, the anticryptococcal activity of NK cells is mediated through a LFA-1-independent pathway [98].

The function of NKT cells has also been shown to be regulated by extracellular pH [99]. These cells are innate-like lymphocytes recognizing lipid antigens and play an important role in the host defense against pathogens and tumor cells. It was reported that extracellular acidosis (pH 6.8) inhibits the production of IFN-*γ* and IL-4 by NKT cells activated by alpha-galactosylceramide. Interestingly, suppression of NKT cell function by low pH was shown to be associated to the inhibition of mammalian target of rapamycin (mTOR) signalling, a critical pathway involved in the activation of both conventional T cells and NKT cells [99].

### 3.4. Dendritic Cells

Conventional or myeloid dendritic cells (DCs) are highly specialized antigen-presenting cells with a unique ability to prime naive T cells inducing the activation of the adaptive immune response [100]. They are responsible for the induction of primary immune responses, but they also play a critical role in determining the type of T cell-mediated immunity as well as in silencing the immune response against self-antigens. DCs do not represent a homogeneous cell population; rather, different functionally specialized subsets of DCs exist, and each of them displays phenotypic and functional plasticity in response to diverse stimuli [101]. The production of fully competent DCs involves two major steps: differentiation of DCs from blood precursors and maturation into potent antigen-presenting cells [102]. Maturation represents a common property of all DC types and subtypes. In fact, upon encountering PAMPs, DAMPs, or inflammatory cytokines in peripheral tissues, DCs become activated and undergo a maturation process leading to an enhanced ability to activate T cells and to direct the differentiation of CD4+ T cells into different profiles. DC maturation is associated with a number of phenotypic and functional changes: (a) upregulation of the chemokine receptor CCR7, allowing homing of DCs to draining nodes drawn by the chemokines CCL19 and CCL21; (b) downregulation of DC ability to capture and process antigens; (c) increased expression of MHC-peptide complexes; (d) upregulation of CD40, CD80, and CD86 expression; and (e) enhanced ability to produce a variety of cytokines, chemokines, and growth factors [103–105].

Extracellular acidosis has been shown to be able to modulate both the differentiation and maturation of DCs. Differentiation of human DCs is usually analyzed by culturing monocytes for 5-7 days with GM-CSF plus IL-4, as described by Sallusto and Lanzavecchia [106]. Studies directed to characterize immune evasion mechanisms in cancer revealed that lactic acidosis impairs the differentiation of monocytes into DCs. Gottfried et al. reported that monocytes cultured in the presence of IL-4 and GM-CSF within multicellular tumor spheroids do not acquire CD1a expression, a marker of monocyte-derived DCs, and show a reduced ability to produce IL-12 [107]. These immunosuppressive effects were shown to be induced by the production of lactic acid by tumor cells. Additional experiments revealed that the inhibitory effect induced by lactic acid could indeed be reverted by adjusting the pH to neutral values, and it was not reproduced by lowering pH with HCl [107], suggesting that the inhibition of DC differentiation depends on lactate transport into the cell, a strictly pH-dependent process [108]. Consistent with this observation, it was reported that monocytes cultured alone at high density, in the presence of IL-4 plus GM-CSF, show impairment in both the acquisition of CD1a expression and the ability to produce IL-12. These immunosuppressive effects were induced by the accumulation of high concentrations of lactic acid in the culture media and were not reproduced by incubating monocytes in culture medium adjusted to low pH values by the addition of HCl [109].

We have studied the influence exerted by extracellular acidosis on the maturation of murine and human DCs. Using DCs derived from murine bone marrow precursors, we found that extracellular acidosis (pH 6.5) increased the endocytic activity of DCs and the expression of MHC class II, CD40, and CD86 [110]. Moreover, DCs pulsed with antigens at low pH values showed an improved efficacy to induce specific cytotoxic responses mediated by CD8+ T cells as well as specific antibody responses in vivo [110]. On the other hand, using human DCs derived from monocytes cultured with IL-4 and GM-CSF, we found that transient exposure of DCs to pH 6.5 markedly increases the expression of HLA-DR, CD40, CD80, CD86, CD83, and CCR7, improves the T cell priming ability of DCs, and increases the production of IL-12, stimulating the synthesis of IFN-*γ*, but not IL-4, by Ag-specific CD4+ T cells [111]. These changes induced by extracellular acidosis were shown to be strictly dependent on the activation of p38 MAPK. Of note, we observed that low concentrations of LPS abrogated DC maturation induced by pH 6.5 [111], suggesting a cross talk between the activation pathways triggered by extracellular protons and LPS in DCs. The ability of extracellular acidosis to induce the phenotypic maturation of human DCs was confirmed by Tong et al. [56]. Interestingly, they demonstrated that this response is induced through the activation of ASIC receptors, a family of proton receptors [56].

### 3.5. Platelets and Endothelial Cells

Different groups have shown that low pH modulates the course of the innate immune response by acting not only on leukocytes but also on nonimmune cells. It is well known that platelets modulate the function of neutrophils in the course of inflammatory processes. Etulain et al. reported that extracellular acidosis (pH 6.5-7.0) downregulates platelet haemostatic functions such as adhesion, spreading, ATP release, aggregation, thromboxane B2 generation, and procoagulant activity, but it increases platelet ability to stimulate inflammatory responses mediated by neutrophils such as chemotaxis and the generation of mixed platelet-leukocyte aggregates, through a P-selectin-dependent mechanism [112]. Low pH has been shown to be also able to modulate the function of vascular endothelial cells [113–117]. Using human umbilical vein endothelial cells (HUVEC), human lung microvascular endothelial cells, and pulmonary artery endothelial cells, it was reported that exposure to pH 6.4 increased the expression of a number of inflammatory genes including chemokines, cytokines, adhesion molecules, COX-2, and NF-*κ*B pathway genes. This proinflammatory effect was induced through the activation of the proton receptor GPR4 [117]. Moreover, it was shown that extracellular acidosis promotes the proangiogenic activity of vascular endothelial colony forming cells and also stimulates lymphoangiogenesis and the production of IL-8 by human lymphatic endothelial cells through a TRPV1-dependent pathway [118].

## 4. Low pH Inhibits T Cell Responses

The abovementioned evidence indicates that extracellular acidosis can either stimulate or suppress innate immune responses depending on both the cell type involved and the particular response analyzed. Contrasting with the diversity of effects induced on the innate immune system, a large body of evidence indicates that low pH strongly suppresses T cell-mediated immunity [119–122]. It has been reported that lactic acid, but not sodium lactate, suppresses the proliferative response and cytokine production by human CD8+ T cells. Inhibition of T cell function by lactic acid was also observed by analyzing infiltrating CD8+ T cells in tumor spheroids [121]. Suppression of T cell function by lactic acid was shown to be a reversible phenomenon which was reversed after a 24 h recovery period in lactic acid-free medium. Interestingly, lactic acid and HCl were shown to induce different patterns of T cell suppression. In fact, lactic acid, but not HCl, markedly suppressed the production of IFN-*γ* and IL-2 by stimulated CD8+ T cells and also promoted cell death. The strong inhibitory effect mediated by lactic acid seems to be mediated, at least in part, via blockade of lactate efflux and thereby disturbance of T cell metabolism [121].

Further supporting that low pH suppresses T cell function, Calcinotto et al. [120] have shown that lowering the environmental pH to values of 6.0-6.5 induced an anergic state in human and mouse tumor-specific CD8+ T cells. This anergic state was characterized by a profound impairment of cytotoxic activity, inhibition of cytokine production, reduced expression of the alpha chain of the IL-2R (CD25), and a diminished activation of extracellular signal-regulated kinase (ERK) and STAT5 upon T cell activation. Buffering pH to neutral values restored T cell function [120, 122]. Of note, raising intratumoral pH with oral sodium bicarbonate in a mice model has been shown to enhance antitumoral T cell responses [122]. Moreover, systemic treatment of tumor-bearing mice with proton pump inhibitors (PPI) improved the therapeutic efficacy of immunotherapy, suggesting that PPI might represent useful therapeutic tools to reverse the anergy of tumor-infiltrating T cells and to improve the performance of immunotherapy approaches used in cancer [120, 123].

The detailed mechanisms through which extracellular acidosis inhibits T cell function have not been yet clarified. T cells are highly dependent on mTORC1 activity in order to cope with their metabolic requirements for activation and differentiation [124, 125]. Balgi et al. reported that low pH inhibits mTORC1 activity in human cell lines [126]. Moreover, in a recent study [127], Walton et al. have shown that low pH markedly suppresses mTORC1 activity in CD4+ and CD8+ T cells through a mechanism dependent, at least in part, on the centrifugal distribution of lysosomes induced by acidosis, impairing the interaction of lysosome-bound mTORC1 with RHEB (GTP-binding protein Ras homolog enriched in brain), an activator of the mTORC1 pathway. These observations suggest that strategies directed to restore mTOR activation in environments characterized by low pH values would represent a useful therapeutic approach in cancer immunotherapy.

An integrated view of the effects induced by extracellular acidosis on the immune response is shown in Figure 1.

## 5. Targeting Acidosis to Improve Cancer Immunotherapy

As mentioned above, extracellular acidosis is associated to the course and severity of autoimmune, allergic, and infectious diseases. Moreover, acidosis represents a hallmark of solid tumors and promotes local tumor progression, metastasis, and resistance to therapy (reviewed in [27, 128]). The contribution of extracellular acidosis to cancer growth is not only related to the suppression of T cell function but also with a number of actions exerted on both tumor cells and the tumor environment. Low pH has been shown to increase cancer cell motility by stimulating cytoskeletal-dependent cancer cell polarization and by increasing the proteolytic activity of TAMs, fibroblasts, and the tumor cells themselves promoting both angiogenesis and cancer invasiveness [129–132]. Moreover, exposure of tumor cells to acidosis results in the induction of autophagy which confers a survival advantage to tumor cells [133]. Considering the role of extracellular acidosis in cancer progression, a large number of studies have analyzed the molecular pathways responsible for acid-base regulation and pH homeostasis in order to improve cancer therapy. The most important pH regulators in tumor cells include different isoforms of carbonic anhydrase and anion exchangers, monocarboxylate transporters, Na^+^/HCO_3_^−^ cotransporters, and Na^+^/H^+^ exchangers [128]. Antibodies directed to these pH regulators and compounds able to modulate its function have been developed and are currently at various stages of clinical development [27, 128]. Moreover, neutralization of tumor acidosis by administration of systemic buffers such as sodium bicarbonate has been shown to hamper tumor growth in experimental models [134, 135]. Interestingly, targeting tumor acidosis has also shown to increase the effectiveness of checkpoint inhibitors (antibodies directed to programmed cell death protein 1 (PD1) and cytotoxic T lymphocyte-associated antigen 4 (CTLA-4)) [120, 122] that have recently demonstrated tremendous potential for the treatment of a variety of solid tumors [136].

## 6. Concluding Remarks

It is widely appreciated that both inflammation and tumor progression are associated with the development of acidic microenvironments. However, there are relatively few studies directed to analyze the effect of extracellular acidosis on the immune response. These studies show that extracellular acidosis suppresses T cell-mediated immunity while it can either stimulate or inhibit the innate immune response, depending on the cell type and the function analyzed. The ability of extracellular protons to activate cell responses mediated by neutrophils, mononuclear phagocytes, DCs, and endothelial cells suggests that a high concentration of protons would be recognized by innate immune cells as a DAMP produced by stressed cells. Supporting this view, it was shown that extracellular acidosis induces cytoplasmic acidification in mononuclear phagocytes inducing IL-1*β* production in a NLRP3-dependent mode. However, other responses triggered by extracellular acidosis seem to be dependent on the recognition of protons by specific pH sensors expressed on the surface of innate cells which are not related to pattern recognition receptors (PRRs). A further complexity derives from the ability of extracellular and intracellular protons to modulate the energetic metabolism of innate immune cells. Acidosis should be understood as an environmental cue arising from stressed tissues where homeostasis has been challenged. Acting in concert with other environment factors, acidosis could be considered as a rheostat of the immune response able to start a proinflammatory or a proresolving immune response depending on the immune context.

## Figures and Tables

**Figure 1:**